# Total and extracellular vesicle–bound plasma P-selectin as diagnostic biomarkers for acute deep vein thrombosis

**DOI:** 10.1016/j.rpth.2025.103171

**Published:** 2025-09-02

**Authors:** Samantha Xavier, Vårin Eiriksdatter Wikan, Casper J.E. Wahlund, Nadezhda Latysheva, Øyvind Øverli, Christopher Antoun, Thor Ueland, Gholamreza Jafari Yeganeh, Sigrid Kufaas Brækkan, John-Bjarne Hansen, Ellen Brodin, Omri Snir

**Affiliations:** 1 Thrombosis Research Group (TREC), Department of Clinical Medicine, UiT – The Arctic University of Norway, Tromsø, Norway; 2 Division of Internal Medicine, Department of Haematology, Akershus University Hospital, Lørenskog, Norway; 3 Thrombosis Research Center (TREC), Division of Internal Medicine, University Hospital of North Norway, Tromsø, Norway; 4 Research Institute of Internal Medicine, Oslo University Hospital, Rikshospitalet, Oslo, Norway; 5 Faculty of Medicine, University of Oslo, Oslo, Norway; 6 Department of Medical Biology, UiT – The Arctic University of Norway, Tromsø, Norway

**Keywords:** P-selectin, DVT, VTE, diagnostic, extracellular vesicles

## Abstract

**Background:**

Plasma P-selectin has shown moderate diagnostic potential for deep vein thrombosis (DVT). In plasma, P-selectin is found in soluble form and bound to extracellular vesicles (EVs). It is unknown whether specific detection of EV-bound P-selectin would improve the diagnostic performance of P-selectin. We aimed to investigate the diagnostic performance of EV-bound and total P-selectin for acute DVT, alone and in combination with D-dimer.

**Objectives:**

We aimed to investigate the diagnostic potential of total extracellular vesicle-bound plasma P-selectin as diagnostic biomarkers for acute deep vein thrombosis in a cohort of patients admitted to hospital with suspected DVT.

**Methods:**

A bead-based flow cytometric assay was developed for selective detection of EV-bound P-selectin. Total and EV-bound P-selectin was measured in 2 cohorts of patients (*n* = 168 and *n* = 200) referred to hospital with suspected DVT. DVT was confirmed or ruled out by compression ultrasound.

**Results:**

DVT was confirmed in 53 patients and ruled out in 115 patients (first cohort) and in 54 and 146 patients (replication cohort), respectively. Only 2% of plasma P-selectin was bound to EVs, and EV-bound P-selectin showed poor diagnostic performance with an area under the receiver-operating characteristic curve (AUC) of 0.55 (95% CI, 0.45-0.65). Total plasma P-selectin had an AUC of 0.70 (95% CI, 0.62-0.78) in the first cohort and 0.77 (95% CI, 0.69-0.85) in the replication cohort. Combination of total P-selectin with D-dimer had diagnostic performance (AUC, 0.88; 95% CI, 0.84-0.91) inferior to D-dimer alone (AUC, 0.92; 95% CI, 0.89-0.95).

**Conclusion:**

The proportion of P-selectin bound to EVs in plasma was low and could not discriminate between patients with and without acute DVT. The diagnostic performance of total P-selectin, alone or in combination with D-dimer, was inferior to the diagnostic performance of D-dimer alone.

## Essentials

• Elevated levels of EVs and P-selectin have been reported in acute DVT.
• This study evaluated the diagnostic performance of EV-bound and total P-selectin for acute DVT.
• Only 2% of total P-selectin in plasma is bound to EVs, and it has poor diagnostic power.
• The diagnostic power of total P-selectin is moderate and does not exceed that of D-dimer.

## 1 Introduction

Diagnosing deep vein thrombosis (DVT) is challenging due to its unspecific signs and symptoms [1]. Clinical guidelines recommend a pretest probability assessment in combination with D-dimer to identify patients in whom DVT can be safely ruled out and patients in whom radiological imaging is required to confirm or rule out the diagnosis [[2], [3], [4]]. However, the pretest probability assessment has some limitations, and studies have revealed poor adherence and incorrect interpretation of this diagnostic algorithm in clinical practice [[5], [6], [7], [8], [9], [10]]. Even with good adherence to the algorithm, only 20% to 35% of those referred to imaging are confirmed to have the disease [11,12], suggesting room for improvement of the algorithm to reduce the use of imaging. Excess diagnostic imaging results in unnecessary use of resources and time, additional costs to patients and health care systems, and, potentially, diagnostic delay [13]. Therefore, diagnostic biomarkers with high specificity for DVT, which can be used alone or in combination with existing diagnostic algorithms, are needed.

P-selectin is a protein expressed on and partially released from activated platelets and endothelial cells [[14], [15], [16], [17]]. Binding of P-selectin to it specific receptor, P-selectin glycoprotein ligand-1, on leukocytes results in initiation of various procoagulant mechanisms including monocyte tissue factor expression, fibrin generation, and formation of highly procoagulant tissue factor–positive extracellular vesicles (EVs) [[18], [19], [20]]. Although several studies have suggested a potential role of soluble P-selectin as a diagnostic biomarker of acute DVT [[21], [22], [23], [24], [25], [26], [27], [28]], many of the studies were limited by unsuitable comparison groups [21,26,28] and small sample size [[21], [22], [23],^25^]. In a diagnostic cross-sectional study of patients with suspicion of proximal lower-extremity DVT, the mean levels of soluble P-selectin were higher in patients with confirmed DVT (*n* = 112) than those in patients without DVT (*n* = 122), and when Wells score was high (≥2 points), P-selectin could rule in DVT, with a higher specificity and positive predictive value than D-dimer [24]. Although this study had a robust sample size, it did not investigate the combination of P-selectin and D-dimer as a diagnostic tool. In a cross-sectional study of 62 patients with acute DVT and 116 patients with leg pain in whom DVT was ruled out, the discriminative performance of P-selectin and D-dimer was similar to that of D-dimer alone with an area under the receiver-operating characteristic curve (AUC) of 0.83 [26].

In plasma, P-selectin can be found as a soluble monomer or bound to EVs [29]. However, the proportion of soluble and EV-bound P-selectin in plasma is currently unknown. Interestingly, higher levels of plasma EVs have been reported in acute DVT [26,30]. Furthermore, the majority of plasma EVs are derived from platelets [31], and higher levels of platelet-derived EVs (which typically carry P-selectin on their surface) are associated with risk of future venous thromboembolism (VTE) [32]. In a study of 22 patients with ultrasound-confirmed DVTs and 21 symptomatic individuals in whom DVT was ruled out, Rectenwald et al. [25] demonstrated that a combination of P-selectin, D-dimer, and total EV count yielded a 73% sensitivity, 81% specificity, and 77% overall accuracy for the diagnosis of DVT. However, they did not distinguish between soluble and EV-bound P-selectin, and to our knowledge, the diagnostic performance of EV-bound P-selectin per se has not been investigated. Currently, methods for high-throughput detection of EV-bound P-selectin are limited.

In this study, we aimed to (i) assess the proportion of EV-bound P-selectin relative to the total P-selectin concentration in plasma from healthy donors, (ii) develop a bead-based assay for high-throughput detection of P-selectin–positive EVs in plasma, (iii) investigate the diagnostic performance of EV-bound P-selectin and total P-selectin for assessment of acute DVT in a cross-sectional study of consecutive patients referred to hospital with suspected DVT, (iv) validate our findings on diagnostic performance of P-selectin in a separate study population, and (v) investigate whether P-selectin in combination with D-dimer could improve the diagnostic algorithm for acute DVT.

## 2 Methods

### 2.1 Preparation of platelet-free plasma from healthy individuals

Platelet-free plasma (PFP) was prepared for assessment of the proportion of EV-bound P-selectin and for validation of the bead-based assay. The study was approved by the Regional Committee for Medical and Health Research Ethics (REK80025). Participants were older than 18 years, did not experience any illness or use medication, and provided a written informed consent. Blood was drawn by venipuncture of the antecubital vein using a 21-gauge needle with minimal stasis. Blood was collected into 9 mL ACD Vacuette tubes (Greiner Bio-One) containing 3.2% sodium citrate of which the first tube was discarded. In addition, 3 mL of blood were drawn into K2EDTA Vacuette tubes (Greiner Bio-One) and used for cell count using an ABX MicrosES60 (ABX Diagnostics). To generate platelet-poor plasma (PPP), blood was centrifuged at 2500*g* for 15 minutes at room temperature using a Heraeus Megafuge 1.0 centrifuge equipped with Heraeus BS4402/A swing bucket rotor. A consecutive centrifugation at 2500*g* for 15 minutes was applied to generate PFP. PFP was aliquoted and frozen at −70 °C until further analysis.

### 2.2 Assessing the proportion of EV-bound P-selectin in plasma

PFP was prepared as described earlier. The soluble protein fraction and the EV fraction in plasma were separated using 2 different methodologies: ultracentrifugation and size-exclusion chromatography (SEC). Ultracentrifugation is a high-yield, yet crude approach to separate nanoparticles from soluble proteins, whereas SEC results in a more accurate separation of the EV and protein fractions. Following fractionation using the 2 approaches, P-selectin concentration was measured by ELISA (i) in unfractionated PFP (representing the total P-selectin concentration in plasma), (ii) in the soluble protein fraction, and (iii) in the EV fraction. The separation of soluble protein and EV fractions from PFP, as well as the identification of the EV fractions separated by SEC, and the measurement of P-selection in such samples by ELISA are described in the supplemental information.

### 2.3 Direct flow cytometry analysis for identification of EVs in fractions of PFP separated by SEC

See Supplementary Methods.

### 2.4 Development and optimization of magnetic bead-based flow cytometry assay to detect P-selectin–positive EVs

A bead-based flow cytometry assay was developed to specifically detect EV-bound P-selectin by using a 2-marker capture and detection system that required the presence of P-selectin as well as a platelet marker (CD41) or a set of EV markers (CD9, CD81, and CD63). The development of the assay and its validation are described in detail in the Supplementary Methods.

### 2.5 Evaluation of total and EV-bound P-selectin as diagnostic biomarkers for acute DVT

#### 2.5.1 Study population and setting

We performed an observational, diagnostic, cross-sectional study of patients with suspected first-time DVT referred to the emergency department or VTE outpatient clinic at Akershus University Hospital (Ahus), Norway, with consecutive inclusion from June 2021 to July 2023. Patient recruited from June 2021 to May 2022 were included in the discovery cohort, while patients recruited from June 2022 to July 2023 composed the replication cohort. This study complies with the Declaration of Helsinki and the Standards for Reporting of Diagnostic Accuracy Studies (STARD) 2015 guidelines [33]. The study was approved by the Regional Committee for Medical and Health Research Ethics (REK 200878). Patients were considered eligible if they had signs and symptoms of DVT (ie, swelling, pain, redness, and venous ectasia), were referred by primary or emergency department physicians to compression ultrasound (CUS) to confirm or rule out DVT, used Ahus as their local hospital, and provided informed written consent to participate in the study. We applied no restrictions regarding pretest probability or D-dimer results for inclusion (ie, all patients were referred to CUS, including those with low pretest probability and D-dimer of <0.5mg/L). Inpatients were not eligible for this study. Patients were excluded if they were <18 or >80 years, had a life expectancy <2 years, were on ongoing therapeutic anticoagulant treatment (except for those receiving a single dose of anticoagulation due to DVT suspicion), or if reference standard (ie, CUS) or index tests (ie, blood collection to measure P-selectin) were not performed or had inconclusive results.

Blood was collected from the study participants at the time of diagnostic workup for suspected DVT (either before or after CUS, but within 24 hours of admission). Blood was drawn from an antecubital vein into vacutainer tubes containing Na-citrate or EDTA using a 21-gauge needle. Blood was centrifuged at 2500*g* for 15 minutes at room temperature and PPP was aliquoted and stored at −80 °C until further used.

All patients were subjected to whole leg CUS examination of the deep veins, which was the reference standard of this study. CUS was performed and interpreted by trained personnel within the first 24 hours after hospital arrival, and the personnel was blinded to the results of the P-selectin measurements. CT venography was not used as reference standard to facilitate direct comparison of the index tests to the diagnostic performance of CUS. A noncompressible distal or proximal deep vein was the diagnostic criterion for presence of DVT, while patients with compressible veins were classified as non-DVT. Patients with recurrent DVT and patients with a first-time DVT requiring catheter-directed thrombolysis were excluded. Patients with DVT and concurrent pulmonary embolism or superficial vein thrombosis were included, whereas patients with isolated pulmonary embolism were excluded. Participants with isolated superficial vein thrombosis were classified as patients without DVT, while patients with intramuscular vein thrombosis were classified as DVT. All patients without DVT were followed up for 3 months after the inclusion date to assess the rate of CUS misclassification.

Information on patient characteristics, DVT symptoms (description and duration), DVT characteristics, comorbidities, differential diagnoses in patients without DVT, as well as results from standard admission blood work (including D-dimer levels) were retrieved by review of medical records. D-dimer was measured by standard immunoturbidimetric methods on Roche Cobas t711 or Cobas t511 using the Tina-quant D-Dimer Gen.2 assay (D-DI2; Roche Diagnostics).

#### 2.5.2 ELISA analysis of total P-selectin and high-sensitivity C-reactive protein in the discovery and replication cohorts

Total P-selectin and high-sensitivity C-reactive protein were measured in PFP by ELISA using commercially available agents according to the manufacturer’s instructions (R&D Systems). The ELISA was performed in a 384-format using the combination of a SELMA pipetting robot (Jena) and a BioTek EL406 dispenser/washer. Absorption was read at 450 nm with a wavelength correction set to 540 nm, using a Synergy H1 Hybrid microplate reader (BioTek). The intraindividual and interindividual coefficients of variation were 3.0% and 8.5% for total P-selectin and 2.6% and 9.1% for high-sensitivity C-reactive protein, respectively.

#### 2.5.3 Bead-based analysis of EV-bound P-selectin

Plasma samples collected from patient (PPP) were thawed in a 37 °C water bath for 2 minutes. Samples were then centrifuged at 2500*g* for 15 minutes to remove platelet remnants and debris, obtaining PFP. Next, samples were diluted 5× in phosphate-buffered saline (PBS) supplemented with 0.1% bovine serum albumin (BSA) and 2 mM EDTA and incubated in rotation with P-selectin capture beads overnight to isolate P-selectin–positive particles in the PFP. Capture beads were washed with PBS/BSA/EDTA buffer thrice. PE-conjugated anti-CD41a antibody and anti-TSPAN antibody were added to the beads to stain platelet-specific EVs and EV-specific markers respectively, and beads were incubated in the dark on ice for 45 minutes. Beads were then washed again with PBS/BSA/EDTA buffer thrice and analyzed by flow cytometry (CytoFLEX; Beckman Coulter). Background signals were determined by comparison with PE-conjugated isotype antibody.

### 2.6 Statistical analysis

Statistical analyses were performed with Stata version 16 (StataCorp LLC), GraphPad Prism 9 (Dotmatics), and R version 4.3.2 (R Core Team). Baseline characteristics were displayed as means with SD or proportions in patients with and without DVT, respectively. The levels of EV-bound P-selectin measured by the bead-based assay (anti-TSPAN and anti-CD41a detection) and the levels of total P-selectin measured by ELISA were displayed in patients with and without DVT using scatter dot plots, and the mean levels were compared in patients with and without DVT using a Mann–Whitney test. An α level of 0.05 was considered statistically significant. To assess the predictive performance, receiver-operating characteristic curves were generated for EV-bound P-selectin (measured by the bead-based assay using anti-CD41a-PE and anti-TSPAN-PE detection) and for total P-selectin (measured via ELISA), and AUC was estimated. These analyses were repeated for the replication cohort. Finally, the 2 cohorts were merged in order to explore whether P-selectin in combination with D-dimer could yield a better diagnostic performance than D-dimer alone. We created a partial dependence plot to explore the relationship between the 2 variables. Furthermore, we created a score where we added the scaled values of the 2 biomarkers together and tested the discriminatory power (ie, the AUC) of the score. We also assessed the AUC of P-selectin in those with a D-dimer level above 0.5 mg/L.

## 3 Results

### 3.1 Assessment of soluble and EV-bound P-selectin in normal human plasma

**Figure 1:**
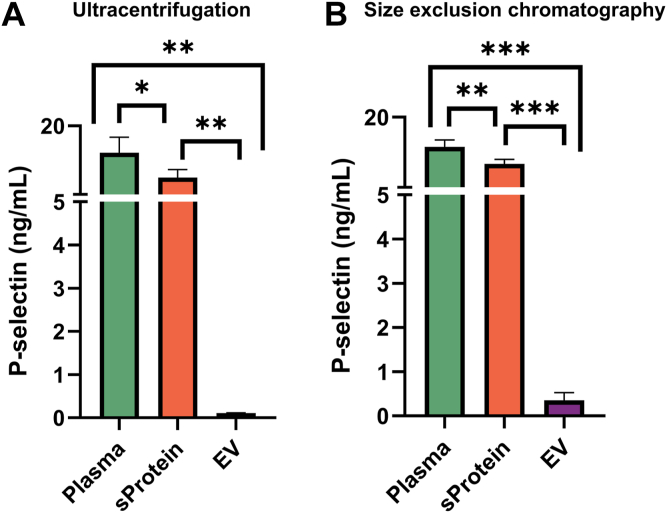
ELISA-based quantification of P-selectin in total plasma, soluble protein (sProtein) fraction, and extracellular vesicle (EV) fraction after ultracentrifugation (A) and size-exclusion chromatography (B) of normal human plasma from 4 healthy donors (∗*P* < .05; ∗∗*P* < .01; and ∗∗∗*P* < .001).

**Supplementary Figure 1:**
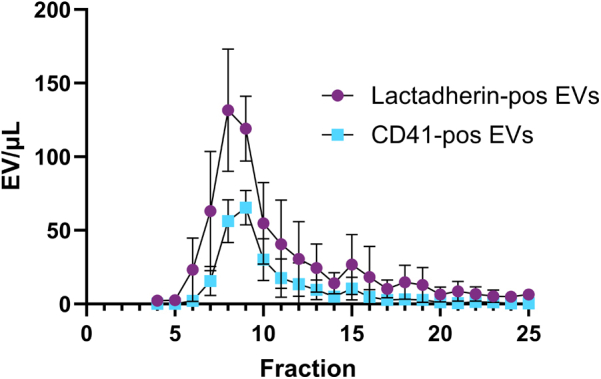
Identification of EV-enriched fractions from SEC-treated PFP (fractions 4-25) from 5 healthy donors. Fractions were stained using Fitc-conjugated lactadherin for detection of phosphatidylserine that is enriched on the surface of EVs and PE-conjugated anti-CD41a antibody for detection of and platelet lineage marker on the EV surface. Following staining, fractions were individually analyzed by flow cytometry. EVs were identified according to size and lactadherin-positive staining (shown in dark purple). EVs that were also positive for CD41 were identified as platelet-EVs (depicted in light blue). Means and error bars for the number of EVs per microliter are presented per SEC fraction.

Figure 1 shows the levels of P-selectin measured by ELISA in unfractionated PFP as well as the soluble protein fraction and in the EV fraction after ultracentrifugation (Figure 1A) and SEC (Figure 1B), respectively. For ultracentrifugation, the mean concentration of P-selectin was 14.13 ± 3.43 ng/mL in plasma, 8.68 ± 1.78 ng/mL in the soluble protein fraction (*P* = .017 for plasma vs protein fraction), and 0.11 ± 0.01 ng/mL in the EV fraction (*P* = .002 for protein fraction vs EV fraction; *P* = .004 for plasma vs EVs fraction) (Figure 1A). After SEC, EVs were detected in fractions 6 to 11, whereas fractions 14 to 25 contained predominantly soluble protein (Supplementary Figure S1). The EV fractions (6-11) and soluble protein fractions (14-25) were separately pooled, and P-selectin was measured by ELISA. The concentration of P-selection was 13.66 ± 1.49 ng/mL in plasma, 10.03 ± 0.98 ng/mL in the soluble protein fraction (*P* = .001), and 0.35 ± 0.18 ng/mL in the EV fraction (*P* < .001 for protein vs EV fraction; *P* < .001 for plasma vs EV fraction) (Figure 1B).

### 3.2 Development of bead-based flow cytometry assay for specific detection of EV-bound P-selectin

**Figure 2:**
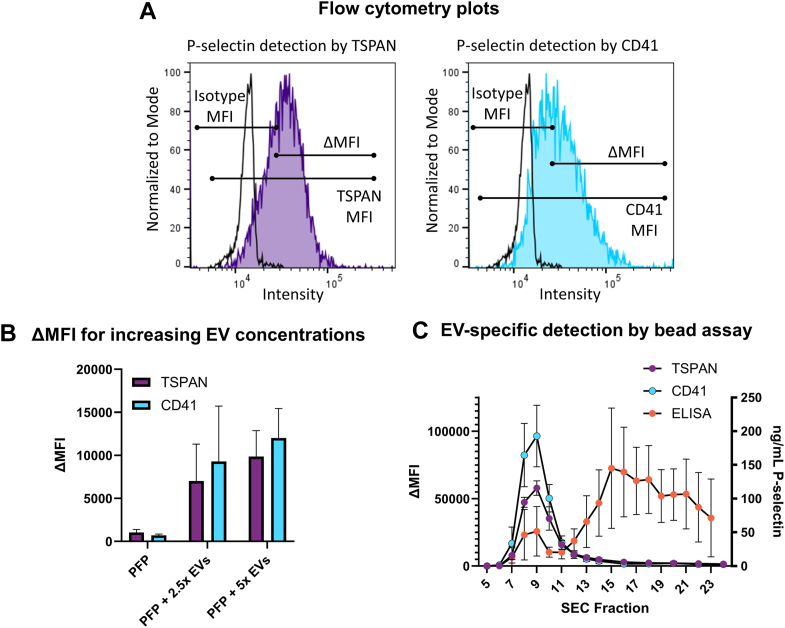
Development of bead-based flow cytometry assay for specific detection of extracellular vesicle (EV)–bound P-selectin. (A) Representative flow cytometry plots of EVs captured on P-selectin Dynabeads and detected by a mix of antitetraspanin (TSPAN) antibodies (ie, CD9, CD63, and CD81) (left) or anti-CD41a (CD41) antibodies (right). Delta mean fluorescence intensity (ΔMFI) values were calculated by subtracting the MFI of the isotype control from the recorded anti-TSPAN or anti-CD41a MFIs. (B) ΔMFI values according to increased amount of P-selectin–positive EVs from activated platelets (2.5× and 5×) spiked into platelet-free plasma (PFP) from healthy individuals (*n* = 3). EVs were captured and detected using anti-TSPAN (purple) and anti-CD41a (blue). (C) ΔMFI values representing EV-bound P-selectin measured by anti-TSPAN (purple) and anti-CD41a (blue) staining of fractions 5-24 of size-exclusion chromatography (SEC)–treated PFP derived from activated platelet-rich plasma in healthy individuals (*n* = 3), and concentration of total P-selectin (orange) in the same SEC-treated PFP as measured by ELISA. EV-bound P-selectin was predominantly detected in fractions 7-10, while fractions 14-24 were enriched with soluble P-selectin.

We developed a bead-based flow cytometry assay to facilitate high-throughput measurement of EV-bound P-selectin. The assay comprised a 2-antibody system, in which EVs carrying P-selectin were selectively captured by SA magnetic Dynabeads coupled to biotinylated monoclonal antibodies specific for P-selectin. The captured EVs were detected by a mix of anti-TSPAN antibodies (ie, CD9, CD63, and CD81), displayed on surface of the majority of EVs, or anti-CD41a, used for detection of platelet-derived EVs. Samples were analyzed by flow cytometry and the mean fluorescence intensities (MFIs) normalized to isotype controls were plotted, as depicted in Figure 2A. Delta MFI (ΔMFI) was calculated by subtraction of the MFI of the respective isotype control.

To test whether increasing levels of P-selectin–positive EVs could be detected by our bead-based assay, PFP samples were spiked with EVs from activated platelets. As shown in Figure 2B, ΔMFI values as detected by anti-TSPAN antibodies were 1036 ± 352 in PFP, 7019 ± 4291 in PFP with 2.5× spiked-in EVs, and 9858 ± 3012 in PFP with 5× spiked-in EVs. The corresponding ΔMFI values as detected by anti-CD41a were 711 ± 141 (PFP), 9285 ± 6446 (2.5× EVs), and 12,028 ± 3411 (5× EVs), respectively.

Lastly, the ability of the bead-based assay to specifically identify P-selectin–positive EVs rather than soluble P-selectin was examined. PRP samples were stimulated using TRAP-6 (to induce the production of platelet-EVs displaying P-selectin), platelets were pelleted, and PFP was prepared and fractionated using SEC. Fractions were analyzed using our bead-based assay as well as by P-selectin ELISA. As shown in Figure 2C, our bead-based assay exclusively detected P-selectin–positive EVs in the EV fractions (Fraction 6-11), whereas no signal was detected in the soluble protein fractions (fractions 14-24). Importantly, ELISA analysis confirmed that P-selectin was present in all fractions and at higher concentrations in the soluble protein fractions.

### 3.3 Diagnostic performance of EV-bound and total P-selectin in acute DVT

**Supplementary Figure 2:**
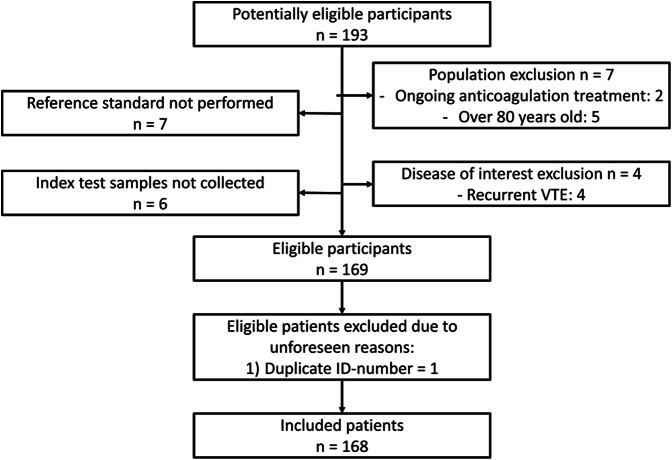
Flowchart of the derivation cohort.

The newly developed bead-based flow cytometry assay and P-selectin ELISA were used to measure EV-bound and total P-selectin levels in plasma from individuals admitted to the emergency room with suspected acute DVT (*n* = 168). Among those, 53 subjects were diagnosed with DVT, while in 115 subjects, DVT was rejected after CUS examination. A flowchart of the study population is displayed in Supplementary Figure S2. The distribution of baseline characteristics of study participants with and without DVT is summarized in Table 1. The mean age was 55 years in patients with confirmed DVT and 59 years in those without DVT. There was a higher percentage of men in the group of patients with DVT (56.6%) than that in those without DVT (45.2%). As expected, the mean D-dimer level was higher in patients with DVT (5.0 mg/L) than that in patients without DVT (0.7 mg/L). Furthermore, CRP levels were higher in those with confirmed DVT (27.5 mg/L) than those in patients without DVT (11.8 mg/L).

**Table 1:** Cohort 1—baseline characteristics of patients with suspected DVT in whom the diagnosis was confirmed (DVT) or ruled out (no DVT) after compression ultrasound.

Characteristic	DVT (*n* = 53)	No DVT (*n* = 115)
Age at VTE (y)	55 ± 15	59 ± 15
Sex (men)	30 (56.6)	52 (45.2)
Overweight (BMI > 25 kg/m^2^)	21 (40.4)	29 (27.1)
Active cancer	3 (5.7)	10 (8.7)
Single-dose anticoagulation	17 (32.1)	25 (21.7)
Duration of symptoms (h)	85.1 ± 24.0	83.1 ± 24.9
Surgery	11 (20.8)	6 (5.2)
White blood cell (10^9^/L)	17.9 ± 52.2	7.6 ± 2.6
Thrombocytes (10^9^/L)	230 ± 79	288 ± 155
Hemoglobin (g/dL)	13.7 ± 2.2	13.8 ± 1.8
D-dimer (mg/L)	5.0 ± 5.3	0.7 ± 0.7
C-reactive protein (mg/L)	27.5 ± 27.8	11.8 ± 21.2
DVT localization		
Proximal DVT	33 (62.3)	–
Distal DVT	19 (35.8)	–
Unspecified	1 (1.9)	–

Values are n (%) or mean ± SD.

BMI, body mass index; DVT, deep vein thrombosis; VTE, venous thromboembolism.

**Figure 3:**
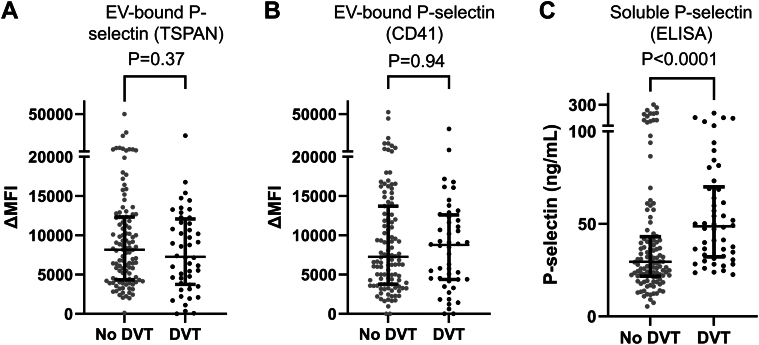
Levels of extracellular vesicle (EV)–bound P-selectin detected by anti-TSPAN (A) or anti-CD41a (B) and total plasma P-selectin (C) in individuals with and without acute deep vein thrombosis (DVT).

**Supplementary Figure 3:**
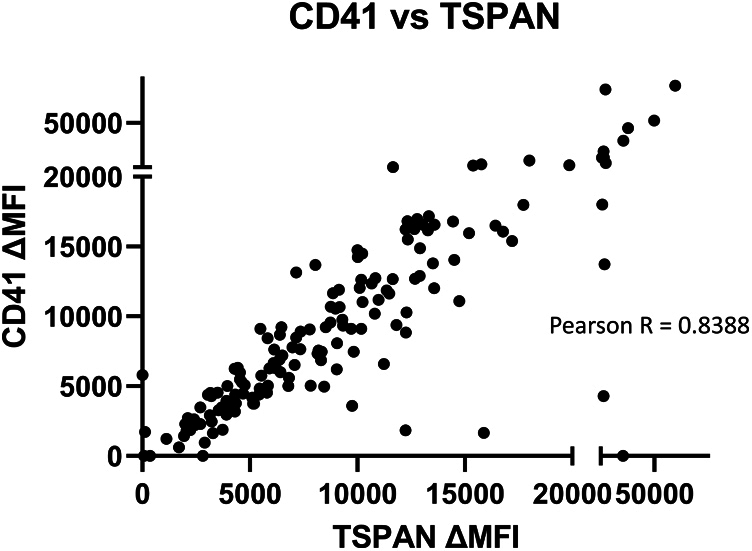
Correlation between EV-bound P-selectin measured by anti-TSPAN-PE and anti-CD41a-PE. Pearson correlation coefficient, R = 0.84, *P* < .0001.

When comparing patients with and without DVT, there were no significant differences in EV-bound P-selectin levels detected with anti-TSPAN (Figure 3A) (ΔMFI, 9178 ± 9672 in the DVT group; 9751 ± 7435 in the no-DVT group; *P* = .37) or anti-CD41a (Figure 3B) (ΔMFI, 10,174 ± 11,682 in the DVT group; 10,183 ± 10,214 in the no-DVT group; *P* = .94). In contrast, the total P-selectin levels measured by ELISA were significantly higher in patients with DVT than those in patients without DVT (61.3 ± 44.3 ng/mL vs 50.8 ± 59.6 ng/mL; *P* < .001) (Figure 3C). Of note, a strong correlation was observed between the levels of EV-bound P-selectin detected by anti-TSPAN and anti-CD41a (Pearson *r* = 0.84) (Supplementary Figure S3).

**Figure 4:**
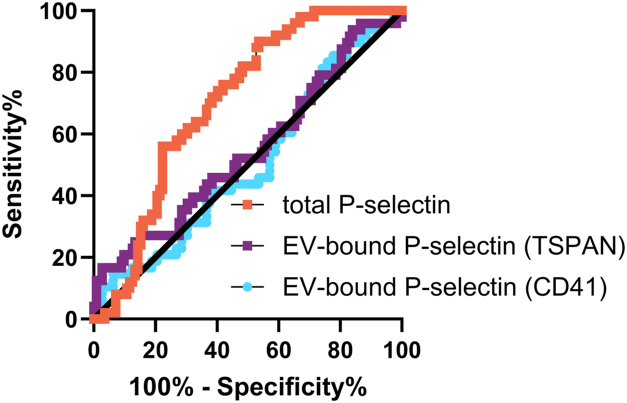
Receiver-operating characteristic curves for diagnosis of acute deep vein thrombosis according to extracellular vesicle (EV)–bound P-selectin detected by antitetraspanin (TSPAN) (purple) or anti-CD41a (blue) using the bead-based assay and total P-selectin (red) measured by conventional ELISA.

The receiver-operating characteristic curves for EV-bound P-selectin (assessed with either TSPAN or anti-CD41a) and total P-selectin are depicted in Figure 4. The AUC was 0.55 (95% CI: 0.45-0.65) and 0.50 (95% CI, 0.41-0.60) for EV-bound P-selectin detected by anti-TSPAN and anti-CD41a, respectively, whereas the AUC for total P-selectin measured by ELISA was 0.70 (95% CI, 0.62-0.78) (Figure 4).

#### 3.3.1 Cohort 2—replication of predictive performance of total P-selectin

**Supplementary Figure 4:**
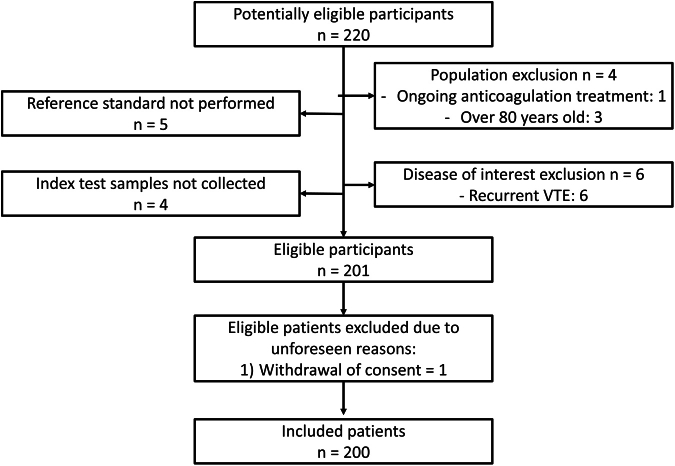
Flowchart of the replication cohort.

The replication cohort (cohort 2) consisted of 54 individuals diagnosed with DVT and 146 individuals in whom DVT was ruled out (Supplementary Figure S4). The distribution of baseline characteristics of study participants is summarized in Table 2. The baseline characteristics of this cohort resembled those of the first cohort, except for a higher proportion of men among patients with DVT (72% in cohort 2 vs 57% in cohort 1).

**Table 2:** Cohort 2—baseline characteristics of patients with suspected DVT in whom the diagnosis was confirmed (DVT) or ruled out (no DVT) after compression ultrasound.

Characteristic	DVT (*n* = 54)	No DVT (*n* = 146)
Age at VTE (y)	58 ± 12	57 ± 14
Sex (men)	39 (72.2)	52 (35.6)
Overweight (BMI > 25 kg/m^2^)	19 (35.2)	37 (26.4)
Active cancer	6 (10.9)	13 (8.9)
Anticoagulation	24 (44.4)	36 (24.7)
Duration of symptoms (h)	81.7 ± 26.5	79.9 ± 30.0
Surgery	7 (13.0)	20 (13.7)
White blood cell (10^9^/L)	11.6 ± 25.4	9.1 ± 20.7
Thrombocytes (10^9^/L)	260 ± 139	275 ± 82
Hemoglobin (g/dL)	14.3 ± 1.8	14.2 ± 1.7
D-dimer (mg/L)	4.7 ± 3.7	0.8 ± 0.6
DVT localization		
Proximal DVT	41 (75.9)	–
Distal DVT	8 (14.8)	–
Unspecified	5 (9.3)	–

Values are n (%) or mean ± SD.

BMI, body mass index; DVT, deep vein thrombosis; VTE, venous thromboembolism.

**Figure 5:**
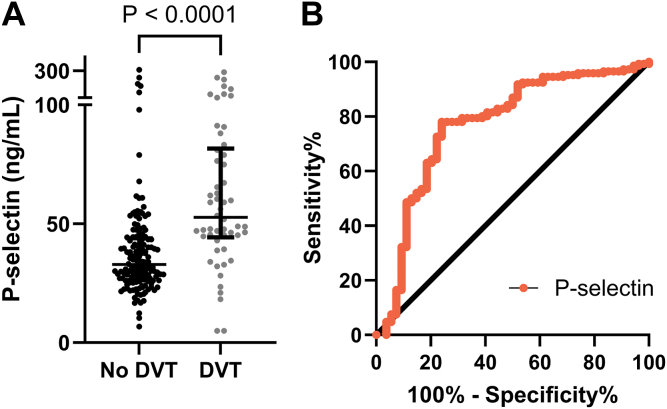
Plasma levels (A) and diagnostic performance (B) of total P-selectin in individuals with and without acute deep vein thrombosis (DVT) in cohort 2 (replication cohort).

Total P-selectin levels measured by ELISA were significantly elevated in patients with DVT compared with those in patients without DVT (71.7 ± 57.2 ng/mL vs 41.4 ± 37.2 ng/mL; *P* < .001) (Figure 5A). The AUC for total P-selectin was 0.77 (95% CI, 0.69-0.85) (Figure 5B).

**Supplementary Figure 5:**
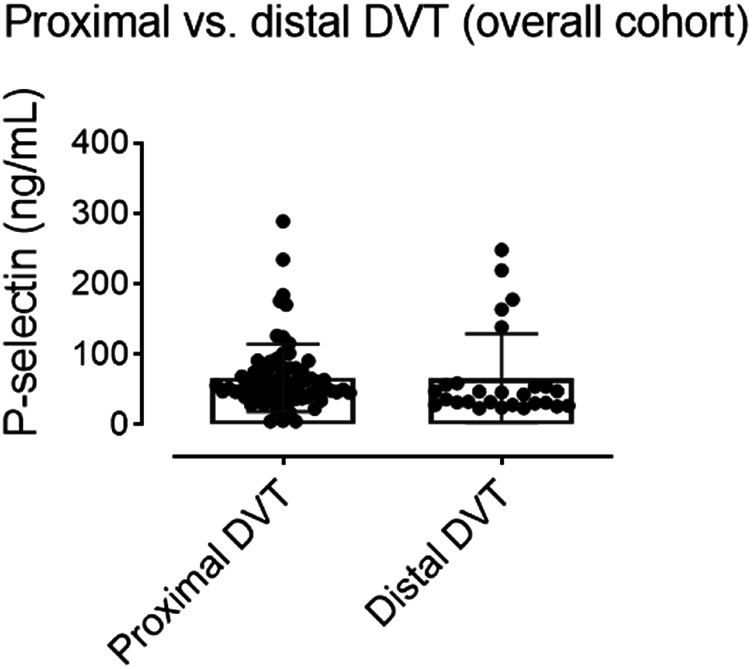
Total P-selectin levels in patients with proximal vs. distal DVT.

**Supplementary Figure 6:**
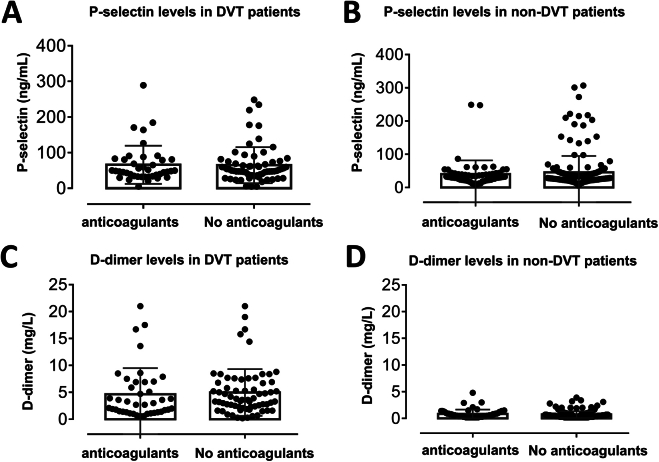
(A) Total P-selectin levels in DVT patients who received vs. did not receive anticoagulants. (B) Total P-selectin levels in patients without DVT who received vs. did not receive anticoagulants. (C) D-dimer levels in DVT patients who received vs. did not receive anticoagulants. (D) D-dimer levels in patients without DVT who received vs. did not receive anticoagulants.

Of note, no significant differences were observed in total P-selectin level between patients with proximal and distal DVTs as measured by a 2-sample *T* test (Supplementary Figure S5) (66.8 ± 5.6 ng/mL vs 66.0 ± 12.2 ng/mL; *P* = .95). Additionally, although some of the study participants received anticoagulant treatment before blood draw, no significant differences were observed in total P-selectin level between patients with DVT who received and those who did not receive anticoagulants (Supplementary Figure S6A) (66.0 ± 8.4 ng/mL vs 64.9 ± 6.2 ng/mL; *P* = .92). Similarly, there were no significant differences in their D-dimer levels (4.6 ± 0.8 mg/L vs 5.0 ± 0.5 mg/L; *P* = .71) (Supplementary Figure S6C).

Likewise, similar levels of P-selectin and D-dimer were found in participants without DVT who were or were not treated with anticoagulants: P-selectin—40.2 ± 5.3 ng/mL vs 45.8 ± 3.5 ng/mL; *P* = .43 (Supplementary Figure S6B); D-dimer—0.84 ± 0.1 mg/L vs 0.72 ± 0.05 mg/L; *P* = .21 (Supplementary Figure S6D).

#### 3.3.2 Combination of total P-selectin and D-dimer for diagnostic performance

**Figure 6:**
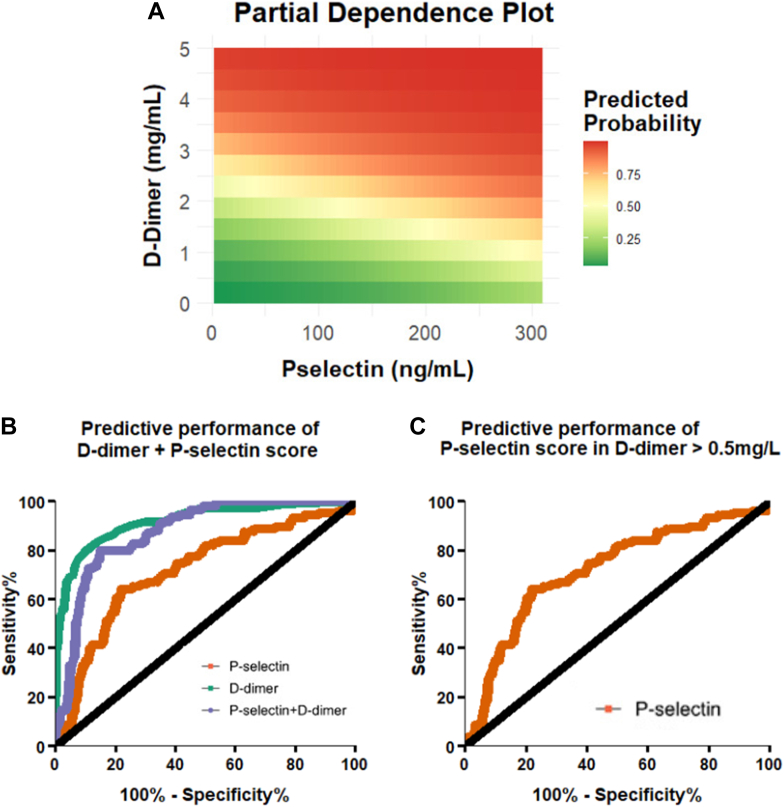
Partial dependence plot for D-dimer and P-selectin for predicted probability of acute deep vein thrombosis (A). Receiver-operating characteristic curve for D-dimer, P-selectin, and a combined score of D-dimer and P-selectin (B). Receiver-operating characteristic curve for P-selectin in individuals with D-dimer >0.5 mg/L (C).

The partial dependence plot showed that the predicted probability of DVT was highly reliant on D-dimer values rather than P-selectin (Figure 6A). Correspondingly, the diagnostic performance of D-dimer and P-selectin (AUC, 0.88; 95% CI, 0.84-0.91) in combination (ie, values added together as a score) was inferior to the diagnostic performance of D-dimer alone (AUC, 0.92; 95% CI, 0.89-0.95) (Figure 6B). In analyses restricted to those with D-dimer of >0.5 mg/L, the AUC of P-selectin was 0.72 (95% CI, 0.67-0.78). When choosing cutoff values to maximize sensitivity and specificity using Youden index, P-selectin (44.69 ng/mL) demonstrated lower sensitivity, specificity, positive predictive value, and negative predictive value (NPV) for acute DVT compared with D-dimer (1.45 mg/L) (Supplementary Table). A previously published cutoff value of P-selectin for DVT diagnosis, 90 ng/mL, was also tested [26]. At 90 ng/mL, P-selectin displayed a sensitivity of 16.36%, specificity of 92.72%, positive predictive value of 0.85, and NPV of 0.73 (Supplementary Table).

## 4 Discussion

In this study, we examined the diagnostic performance of EV-bound P-selectin and total plasma P-selectin for the diagnosis of acute DVT. We found that the proportion of EV-bound P-selectin in plasma was as low as 2% of the total P-selectin. We successfully developed a bead-based assay that enabled high-throughput accurate and specific detection of EV-bound P-selectin, and we applied this assay on samples from a cross-sectional study of patients admitted to an emergency room with suspected DVT. We found that EV-bound P-selectin was no better than chance at discriminating between individuals with and without acute DVT as the AUC was 0.50-0.55. In contrast, total P-selectin showed moderate diagnostic performance with an AUC of 0.70 in the first cohort and 0.77 in the replication cohort. However, the combination of P-selectin and D-dimer did not exceed the diagnostic performance of D-dimer alone for the diagnosis of acute DVT.

P-selectin is a single-chain glycoprotein stored in the α-granules of platelets and the Weibel-Palade bodies in endothelial cells [[14], [15], [16], [17]]. Upon activation, the protein is rapidly translocated and expressed on the surface of these cells and further released in its soluble form into plasma via proteolytic cleavage [[34], [35], [36]]. In addition, P-selectin in plasma is also found on EVs released from activated platelets [37] and endothelial cells [38]. To the best of our knowledge, this work is the first to investigate the proportions of soluble and EV-bound P-selectin in total plasma. We found that approximately 2% of all P-selectin in plasma is bound to EV, indicating that upon cell activation, P-selectin is predominantly released in its soluble form via proteolytic cleavage.

Previous studies have shown higher levels of plasma EVs in patients with VTE compared with those of healthy controls [39,40] and evaluated their potential as diagnostic markers in acute VTE [25,41]. Rectenwald et al. [25] compared 22 patients with ultrasound-confirmed DVT and 21 symptomatic individuals in whom DVT was ruled out and reported that a combination of P-selectin, D-dimer, and total EV count yielded an AUC of 0.79 for the diagnosis of DVT. Individually, the total EV count had low diagnostic performance for VTE (AUC, 0.61), while P-selectin showed an AUC of 0.75 [25]. Similarly, Ramacciotti et al. [26] reported that total EV count did not discriminate between patients with and without DVT, while P-selectin had an AUC of 0.76, in a study of 62 patients with DVT and 116 in whom DVT was ruled out [26]. Notably, the authors did not distinguish between soluble and EV-bound P-selectin in their assessment of diagnostic performance in these studies [25,26]. Our study showed that direct measurement of EV-bound P-selectin had no discriminatory power for acute DVT. The lack of diagnostic utility could likely be explained by the low proportion of EV-bound P-selectin compared with the total P-selectin concentration present in plasma. Alternatively, platelet and/or endothelial cell activation with subsequent release of EV-bound P-selectin could be a key feature of DVT as well as other conditions with similar clinical manifestations (differential diagnosis) and thereby not be useful to discriminate between diagnoses. Finally, potential inaccuracy in quantification of EV-bound P-selectin, leading to a dilution of our results, cannot be completely ruled out.

Previous studies on the role of P-selectin in DVT diagnosis have indicated that P-selectin has moderate predictive potential for DVT, yet many of these studies have been limited by unsuitable comparison groups [21,26,28] and small sample sizes [[21], [22], [23],^25^]. Our findings are in line with previous studies with a comparable design, which reported AUC values for P-selectin ranging from 0.75-0.84 [23,25,26]. Few studies have assessed the diagnostic performance of plasma P-selectin in combination with D-dimer. In the study by Rechtenwald et al. [25], the combination of P-selectin, D-dimer, and total EV count yielded a higher AUC (0.79) than P-selectin (AUC 0.75) and D-dimer (0.71) individually (the AUC for P-selectin and D-dimer combined was not reported). In the study by Ramacciotti et al. [26], the combination of P-selectin (cutpoint, 90 ng/mL) and D-dimer (cutpoint, 0.5 mg/L) yielded an AUC of 0.83, which was similar to that of D-dimer alone (AUC, 0.83). In agreement with their findings, we found that P-selectin displayed moderate discriminatory performance but was still inferior to D-dimer, indicating that P-selectin cannot replace or supplement D-dimer to improve the diagnostic algorithm.

Our study design provides a robust analysis of the diagnostic potential of P-selectin by mirroring clinical reality. We included consecutive patients referred to the outpatient DVT clinic or emergency department at Ahus Hospital with suspected DVT, without excluding patients with negative D-dimer or patients without DVT based on differential diagnoses. We also did not require pretest probability assessments (eg, Wells Score) to be conducted before inclusion in our study, as our interest was to discover a biomarker that could discriminate well regardless of pretest probability. Nonetheless, we observed a DVT prevalence of 30% in our study population, which is similar to that reported in other consent-based clinical studies [26], but slightly higher than the reported prevalence of approximately 15% to 20% among completely unselected patients referred to hospital emergency rooms with suspected DVT in Norway [12,28,42]. This is likely explained by a slightly lower recruitment rate among patients without DVT as they leave hospital earlier or can be more likely to decline study attendance. Since the NPV is dependent on the prevalence of disease, the NPV of D-dimer as a standalone test was somewhat lower in our study (97.3%) than the NPV observed in other studies (98%-99%) [12,43]. The use of CUS, which has slightly lower sensitivity than CT venography (gold standard) for detection of DVT could potentially have introduced some misclassification of DVTs. However, the application of whole leg rather than proximal CUS likely increased the sensitivity of the examination, and the degree of misclassification is expected to be low. Of note, 3 DVTs and 1 PE were observed among those who were initially classified as no DVT during the 3 months of follow-up, and it is unlikely that this misclassification has influenced the results. Approximately 24% of all patients received a single dose of anticoagulation before CUS and blood sampling in our study, presumably due to high suspicion of DVT and delayed CUS. While this is normal clinical practice in Norway (particularly if a patient is referred to hospital in the evening and CUS cannot be performed until the next morning), it may potentially impact the diagnostic accuracy of soluble and EV-bound P-selectin. While studies have not directly assessed whether plasma P-selectin levels are influenced by LMWHs or direct oral anticoagulants in particular, it has been suggested that P-selectin levels in patients with DVT are lowered following treatment with vitamin K antagonists [28]. Furthermore, heparins have been shown to interfere with P-selectin mediated cell adhesion [44]. If administration of a single-dose low-molecular-weight heparin or direct oral anticoagulant contributed to lower P-selectin levels on an individual level, this could potentially have diluted our results, as the proportion receiving anticoagulants was somewhat higher among those with acute DVT than that in among those without. Importantly, there were no significant differences in total P-selectin and D-dimer levels between patients with and without DVT who received and did not receive a bolus of anticoagulants before blood collection. To pursue a high-throughput assay that could easily be applied in the clinical setting, we did not perform any preanalytical processing of the plasma samples. Although we showed that our bead-based assay was specific for detection of EVs and could detect differential levels of EV-bound P-selectin in plasma, the possibility of nonspecific binding of other plasma proteins to the beads, leading to a dilution of our results, cannot be ruled out. Potentially, preisolation of plasma EVs by SEC fractionation or other approaches could have improved the specificity of the bead-based assay. It is important to note that the results of our study predominantly apply to people of White descent.

In conclusion, we successfully developed a bead-based assay for high-throughput detection of EV-bound P-selectin in plasma. We found that the proportion of P-selectin bound to EVs in plasma was very low and that EV-bound P-selectin could not be used to discriminate patients with and without acute DVT among patients referred to the emergency room or outpatient clinic with suspicion of DVT. Total P-selectin levels in plasma displayed moderate diagnostic performance for acute DVT in both the initial and the replication cohorts. However, total P-selectin alone or in combination with D-dimer was inferior or equal to the diagnostic performance of D-dimer alone and could not be used to improve the diagnostic algorithm for DVT, respectively.

## Figures and Tables

**Supplementary Table 1:**
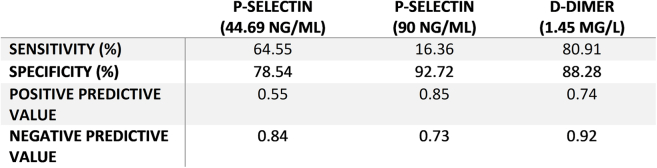
Sensitivity, specificity, positive and negative predictive values for total P-selectin and D-dimer in the overall cohort with cutoffs chosen through the maximization of Youden’s Index and previous literature.
