# Supplementary material for: Total and extracellular vesicle–bound plasma P-selectin as diagnostic biomarkers for acute deep vein thrombosis
Source: Res Pract Thromb Haemost. 2025 Sep 2;9(7):103171. doi: 10.1016/j.rpth.2025.103171 (PMC12514487; doi:10.1016/j.rpth.2025.103171)
Supplement: Supplementary Material [file mmc1.docx]

**Supplementary Methods**

- - 1. **Separation of PFP to EV and soluble protein fractions by ultracentrifugation**

Frozen PFP samples (500 μl) were thawed for 2 minutes in a 37°C water bath. The samples were loaded into Ultra-Clear™ Centrifuge tubes (Beckman Coulter), diluted 25x in PBS and centrifuged at 100,000 xg for 1 hour at 5°C. The supernatants (i.e., soluble protein fraction) were transferred into separate tubes and the EV pellets were resuspended in 250 μl PBS. Both fractions, EV and soluble protein, were immediately analyzed for the levels of P-selectin using ELISA as described in 2.2.3.

- - 1. **Fractionation of PFP to EVs and soluble protein using size exclusion chromatography**

Filtration columns (15 mL, TELOS, Queensland, Australia) loaded with 10 ml of SEPHAROSE CL2B and fitted with polyethylene frits (TELOS, Queensland, Australia) were washed once with 1x column volume phosphate buffered saline (PBS, Sigma-D8537) at room temperature. Next, 500 µL PFP was thawed for 2 minutes in a 37°C water bath and PBS was added to a final volume of 1 mL, resulting in a 2x dilution of the plasma. The diluted PFP (1 mL) was applied to the size exclusion chromatography columns and 25 fractions of 500 µL each were collected following a continuous addition of PBS to the columns. A 70 μL aliquot was collected from each fraction from 5-25 and kept at -70°C until further analysis of total P-selectin by ELISA (described in detail in 2.2.3). The remaining volume was used to determine the presence of EVs by flow cytometry, as described below in 2.4.1.

- - 1. **Analysis of EV-bound and soluble P-selectin concentration in human plasma**

The EV and soluble protein fractions separated by ultracentrifugation (2.2.1) as well as the individual fractions derived from size-exclusion chromatography (2.2.2) were analyzed for P-selectin concentration using a commercially available P-selectin ELISA kit (R&D Systems, DY137), according to the manufacture’s protocol.

Supernatants from PFP samples separated by ultracentrifugation were analyzed by ELISA in a 100x dilution (in PBS) and the EVs pellets were concentrated 2x using Ultracel-10K filters (Merck Millipore, Tullagreen, Ireland) (i.e., EVs from 500 μl PFP resuspended in 250 μl PBS). Fractions collected by size-exclusion chromatography were combined to an ‘EV pool’ (fraction 6-11) and a ‘soluble protein pool’ (fraction 14-25), based on the lactadherin/anti-CD41a analysis described in supplementary methods. Fractions 1-5 were excluded from further analysis as fractions 1-4 were dead volume and fraction 5 did not contain detectable EVs or P-selectin via CD81/CD9/CD63 and CD62P staining respectively followed by flow cytometry. An unfractionated plasma sample (i.e., PFP) from each of the donors was analyzed at both a 25x and 100x dilution. All samples were measured in duplicates, and optical densities (OD) were determined at 450 nm using a microplate reader (Multiskan™ FC, ThermoFisher Scientific). A standard curve was generated using a serial dilution of a P-selectin standard at a set concentration provided by the manufacturer and used to determine the concentration of P-selectin in each sample. The average concentrations of P-selectin in the EV fractions, protein fractions

- 1. ***Direct flow cytometry analysis for identification of EVs in fractions of PFP separated by size-exclusion chromatography***

PFP fractions separated by size-exclusion chromatography were analyzed by flow cytometry to confirm the presence of EVs and to identify the fractions containing EVs. Fractions were double-stained with FITC-conjugated lactadherin (Haematologic Technologies), which is highly specific for the phosphatidylserine displayed on the outer leaflet of the EV membrane, and PE-conjugated anti-human CD41a antibody (HIP8, Biolegend) for detection of platelet-specific EVs. Lactadherin-FITC (2 μL per sample) and anti-CD41a (1 μL per sample) along with PBS (7 μL per sample) were filtered using a 0.22µM ultrafree MC column (Merck, County Cork, Ireland) before being added to the size-exclusion chromatography fractions and incubated in the dark on ice for 45 minutes. Next, PBS was added to the samples up to a volume of 1 mL, and samples centrifuged at 20,000 xg for 30 min at 5°C to remove unbound lactadherin or anti-CD41a antibody. The EV pellets were re-suspended in 200 μL PBS and samples were analyzed using CytoFLEX (Beckman Coulter) at a rate of 10 μL/min. Data analysis was performed using CytExpert 2.0 (Beckman Coulter).

- 1. ***Development and optimization of magnetic bead-based flow cytometry assay to detect P-selectin positive EVs***

A bead-based flow cytometry assay was developed to specifically detect EV-bound P-selectin by utilizing a two-marker capture and detection system that required the presence of P-selectin as well as a platelet marker (CD41) or a set of EV markers (CD9, CD81, and CD63).

- - 1. **Capture and detection protocol in the magnetic bead-based assay**

A monoclonal anti-P-selectin antibody (clone AK4, Biolegend) was biotinylated in-house using EZ-Link™ Sulfo-NHS-Biotin No-Weigh™ Format (ThermoFisher Scientific) and conjugated to streptavidin-coated magnetic beads (Dynabeads™ M-280 Streptavidin, ThermoFisher Scientific), hereafter referred to as capture beads. Capture beads (1 mg/mL) were added to PFP diluted 5x in PBS and incubated overnight at 4°C in order to facilitate binding of P-selectin-positive particles in the PFP. Following incubation, the capture beads were washed in PBS supplemented with 0.1% bovine serum albumin (BSA) and 2 mM EDTA and stained with either (i) a mix of anti-tetraspanin (TSPAN) antibodies composed of CD9-Phycoerythrin (PE) (clone HI9a, Biolegend), CD63-PE (clone H5C6, Biolegend), and CD81-PE (clone 5A6, Biolegend), or (ii) anti-CD41a-PE antibody (clone HIP8, Biolegend) to detect EV-bound P-selectin targeted by the P-selectin capture beads. Of note, CD9, CD63, and CD81 are displayed on the surface of the majority of EVs while CD41a is specifically displayed on the surface of EVs produced by platelets. Samples were incubated in the dark on ice for 45 minutes, washed with a PBS/BSA/EDTA buffer and mean fluorescent intensity (MFI) was acquired by flow cytometry (CytoFLEX, Beckman Coulter). The background signal was determined by staining with PE-conjugated isotype control. Data was analyzed by FlowJo V.10.

- - 1. **Validation of bead-based flow assay using plasma with spiked-in EVs**

To test our bead-based flow cytometry assay ability to detect changes in the concentration of EVs in plasma, P-selectin-positive EVs from washed platelets were spiked into PFP prepared from healthy individuals as previously described in [33].

EVs from 125x10^6^ TRAP-activated platelets spiked into PFP to 500 µL PFP were considered to be 2.5x EVs, while EVs from 250x10^6^ TRAP-activated platelets added to 500 µL PFP were considered to be 5x EVs. Control PFP along with PFP with spiked-in EVs from activated washed platelets were incubated overnight with P-selectin capture beads and analyzed using flow cytometry by a mix of anti-TSPAN antibodies, anti-CD41a-PE, and a PE-conjugated isotype control as described in 2.4.1.

Delta MFI was calculated by subtracting the MFI given by the PE-conjugated isotype from the MFI given by the antibody-stained sample and plotted in a bar graph using GraphPad Prism 9 (Dotmatics, Massachusetts, USA).

**2.4.3 Specificity of the bead-based flow assay to detect EV-bound rather than soluble P-selectin**

To assess the ability of the bead-based assay to detect EV-bound rather than soluble P-selectin, both fractionated and whole PRP from healthy individuals were analyzed with/without stimulations to generate P-selectin positive EVs. Blood was collected from 3 healthy donors and PRP was generated by centrifugation at 140 xg for 15 minutes with no breaks. PRP was activated with 100 µM TRAP-6 for 15 minutes in a 37°C water bath in order to stimulate platelets to release P-selectin-positive EVs. EDTA (0.5M – diluted to a final concentration of 10mM) was added to all tubes to terminate activation, and platelets were pelleted by centrifugation at 2,500 xg for 15 minutes to generate PFP. PFP samples were fractionated by SEC as described in 2.2.2. Fractions 5-24 were analyzed for P-selectin content by the magnetic bead-based flow cytometry assay (as described in 2.4.1) and with P-selectin ELISA, as described in 2.2.3.

Delta MFI and P-selectin concentration in ng/mL were plotted as means ± standard deviation in GraphPad Prism 9 (Dotmatics, Massachusetts, USA). Delta MFI was calculated and plotted for each fraction and concentration of protein in ng/mL was calculated based on a standard curve obtained from ELISA on a line graph as a representation of means ± standard deviation. Because the bead-based assay and ELISA both measure P-selectin concentration but do so in different units, two separate y-axes were generated for the same graph in order to better compare results from the two different methods.
